# etymologia: ***Chimera* (ki-mir′ə)**

**DOI:** 10.3201/eid1411.999999

**Published:** 2008-11

From the Greek *Khimaira,* Latin *Chimaera*; she-goat. In Greek mythology: a composite creature with the body and head of a lion, a goat’s head rising from its back, and a serpent’s tail. In science: an individual organism whose body contains cell populations derived from different zygotes, of the same or different species. Each population of cells keeps its own character, and the resulting animal is a mixture of tissues. Chimera also refers to a substance created from proteins or genes of 2 species, as by genetic engineering. Chimerism is rare in humans; ≈40 cases have been reported.

